# Development of a Nomogram to Predict Disease-Specific Survival for Patients After Resection of a Non-Metastatic Adenocarcinoma of the Pancreatic Body and Tail

**DOI:** 10.3389/fonc.2020.526602

**Published:** 2020-10-29

**Authors:** Yiping Zou, Hongwei Han, Shiye Ruan, Zhixiang Jian, Liang Jin, Yuanpeng Zhang, Zhihong Chen, Zi Yin, Zuyi Ma, Haosheng Jin, Menghua Dai, Ning Shi

**Affiliations:** ^1^Department of General Surgery, Guangdong Provincial People’s Hospital, Guangdong Academy of Medical Sciences, Guangzhou, China; ^2^Shantou University Medical College, Shantou, China; ^3^The Second School of Clinical Medicine, Southern Medical University, Guangzhou, China; ^4^Department of General Surgery, Peking Union Medical College Hospital, Chinese Academy of Medical Sciences and Peking Union Medical College, Beijing, China

**Keywords:** pancreatic body and tail adenocarcinoma, risk factors, prognosis, nomogram, SEER database

## Abstract

**Background:**

Models for predicting patient survival after resection of a non-metastatic adenocarcinoma of the pancreatic body and tail (APBT) are scarce. We wished to establish and validate a nomogram to predict disease-specific survival (DSS) of these patients.

**Methods:**

A total of 1,435 patients screened from the Surveillance, Epidemiology, and End Results (SEER) database were included and divided randomly into a training set (TS; *n* = 1,007) and internal validation set (IVS; *n* = 428) at a ratio of 7:3. Cox regression analyses were conducted to select independent predictors in the TS, and a nomogram was constructed. The model was subjected to the IVS and an external validation set (EVS) comprising 151 patients from two tertiary hospitals.

**Results:**

Five independent risk factors (age at the diagnosis, chemotherapy, tumor grade, T stage, and the lymph node radio) were identified and integrated into the nomogram. Calibration curves indicated that the nomogram could predict DSS at 1, 2, and 3 years accurately. The nomogram had a higher concordance index for predicting DSS compared with that using the 8th edition of the American Joint 23 Committee on Cancer (AJCC8) stage (TS: 0.681 *vs*. 0.606; IVS: 0.662 *vs*. 0.590; and EVS: 0.675 *vs*. 0.608). The nomogram had better discrimination ability and clinical utility than the AJCC8 stage for predicting 1-, 2-, and 3-year DSS.

**Conclusion:**

Our developed nomogram could accurately predict DSS in patients after resection of a non-metastatic APBT.

## Introduction

Pancreatic cancer is one of the most lethal malignant tumors worldwide. Pancreatic adenocarcinoma is the most common pathologic type, accounting for 85% of all types of pancreatic cancer. In 2018, the number of newly diagnosed cases of pancreatic cancer worldwide was 458,918 cases, with 432,242 patients dying (1). In the United States, pancreatic cancer is the 11th most common cancer and the third leading cause of cancer-related mortality, with a 5-year survival of 8.5% (2).

Resection is the only potentially curative therapy for pancreatic cancer. However, due to a lack of obvious symptoms and reliable biomarkers, 35% of patients are diagnosed at a locally advanced (III) stage, and 50% are diagnosed at the metastasis (IV) stage. Therefore, most of these patients do not have the opportunity to undergo resection (3).

In pancreatic cancer, the location of the tumor is an important risk factor. Several studies have demonstrated that, compared with an adenocarcinoma of the pancreatic head (APH), an adenocarcinoma of the pancreatic body and tail (APBT) carries a lower possibility of resection and worse survival (4–6). These different outcomes are attributed largely to a later diagnosis and suboptimal studies of molecular pathology.

Usually, the first symptom of a tumor located in the head of the pancreas is obstructive jaundice, which can be detected early. In contrast, a tumor located in the body and tail of the pancreas cannot be diagnosed until it has reached an advanced stage with the late-appearing symptoms of cachexia and pain (7). At the molecular level, tumors located in the body and tail of the pancreas have richer genetic programs involved in tumor invasion and epithelial-to-mesenchymal transition (8). A tumor located in the pancreatic head can be resected by pancreaticoduodenectomy. Typically, a tumor in the body or tail of the pancreas is treated by distal pancreatectomy combined with splenectomy (9), which is associated with a poor short-term outcome (10).

The prognosis of pancreatic cancer is assessed using the eighth edition of the American Joint Committee on Cancer (AJCC) staging system (“AJCC8”). This is based on the size and extent of the tumor (T), the number (N) of positive lymph nodes (N), and distant metastasis (M) of the tumor (11). However, Allen and colleagues indicated the need for improved prognostic tools (12).

A “nomogram” is a useful statistical model. A nomogram can be used to predict the individual oncologic prognosis by combining relevant factors (13). Nomograms after resection of an APH have been reported (14, 15), but not for an APBT. Due to their heterogeneity, it is necessary to create a nomogram to predict the prognosis of patients after resection of an APBT.

We aimed to identify the independent factors of disease-specific survival (DSS) in patients after resection of a non-metastatic APBT. In this way, we wished to establish a nomogram to predict the DSS of these patients. This scoring system could help clinicians to reach a more appropriate clinical decision and to identify patients at high risk of recurrence and disease progression after surgery.

## Materials and Methods

### Ethical Approval of the Study Protocol

The study protocol was approved by the Ethics Research Committee of Guangdong Provincial People’s Hospital within the Guangdong Academy of Medical Sciences (Guangdong, China) and Peking Union Medical College Hospital within the Chinese Academy of Medical Sciences (Beijing, China). This clinical study was undertaken according to the Declaration of Helsinki 1964 and its later amendments.

### Study Population

We collected (retrospectively) data from the Surveillance, Epidemiology, and End Results (SEER) database and two tertiary hospitals in China. The data source of 1,435 patients with a resected APBT was acquired using SEER^∗^Stat v8.3.5.^1^ We obtained permission in November 2018 to analyze research data (Username: 14376–Nov2018).

The study cohort consisted of patients with International Classification of Diseases for Oncology, Third Edition (ICD-O-3) histology codes of 8140, 8255, 8480, 8481, 8500, 8521, and ICD site codes C25.1 and C25.2.

The inclusion criteria were patients (i) aged >18 years and diagnosed with a pancreatic adenocarcinoma between 2004 and 2015; (ii) with no history of other malignant tumors (sequence number: one primary only; first malignant primary indicator: yes); and (iii) with an “other cause of death” classification from SEER (alive or dead due to cancer).

The exclusion criteria were patients (i) with missing or incomplete information about therapies, follow-up, tumor sizes, and other characteristics; (ii) who underwent non-operative treatment; (iii) for whom the follow-up month was 0 (perioperative death); (iv) who did not have a pathologic diagnosis; (v) whose lymph nodes were not examined; and (vi) with distant metastasis (unconventional surgical indication).

The variables we analyzed were age at the diagnosis, sex, ethnicity, primary site of the tumor, tumor Grade, radiotherapy, chemotherapy, marital status at the diagnosis, T stage, and lymph node ratio (LNR). The *T* stage according to AJCC8 was calculated based on tumor size and vascular invasion. This information, together with the number of positive lymph nodes, was used to calculate the AJCC8 stage. The LNR was obtained by dividing the number of lymph nodes examined by the number of positive lymph nodes. “DSS” was defined as the time from the clinical diagnosis to cancer-related death.

Patient demographics (age at the diagnosis, sex, and ethnicity) and disease features of tumors (size, site, histology grade, *T* stage, AJCC8 stage, radiotherapy, chemotherapy, and the LNR) at baseline were collected from the SEER database. For nomogram construction and internal validation, patients from the SEER database were divided randomly into a training set (TS; *n* = 1,007) and internal validation set (IVS; *n* = 428) at a ratio of 7:3. The TS was used to create a nomogram to predict DSS in patients after resection of a non-metastatic APBT. Then, the IVS was used to evaluate the benefit of this novel model.

**FIGURE 1:**
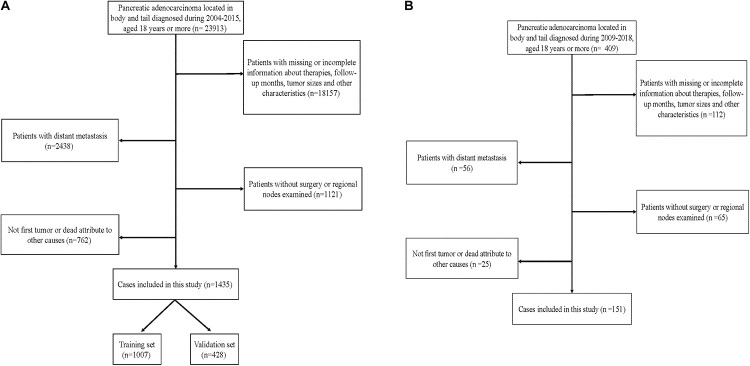
Flowchart showing selection of the **(A)** Surveillance, Epidemiology, and End Results (SEER) database and **(B)** external cohort.

To examine the generalizability of our model, an external validation set (EVS) of patients was provided. This cohort comprised 151 patients diagnosed with an APBT and who underwent surgery between 2009 and 2018 in the Peking Union Medical College Hospital or Guangdong Provincial People’s Hospital. The inclusion criteria and exclusion criteria for these data were consistent with those in the SEER database. Figure 1 shows the data-screening process of the SEER database and our EVS.

### Statistical Analyses

The best cutoff points of age at the diagnosis and the LNR were calculated using X-tile (Yale School of Medicine, New Haven, CT, United States) for outcome-based optimization. With regard to patient demographics at baseline as well as the disease features of the TS, IVS, and EVS, categorical variables are expressed as percentages. Cox proportional hazards multivariable regression was used to identify the independent effects of the univariate prognostic factors on DSS using R v3.6.1.^2^ Hazard ratios (HRs) and 95% confidence intervals (CIs) were computed based on the Cox regression analysis. *P* < 0.05 (two-sided) was considered significant.

A nomogram was constructed based on the result of the multivariate Cox regression model using the “rms” program within *R*. Then, the scores of all the variables were calculated. Each patient received his/her total number of points from the nomogram (“Nomo-score”). The TS was divided into three risk groups (“low,” “medium,” and “high”) according to the sum-score of each patient on the nomogram. The cutoff points of risk stratification were calculated by X-tail software. The Kaplan–Meier survival curves of the three risk groups were plotted separately. *P* < 0.05 was considered significant. To evaluate the discrimination ability of the nomogram, we used the concordance index (C-index) and the area under the receiver-operating characteristic curve (AUC). To compare the association between the actual outcomes and predicted probabilities, calibration curves were used. The receiver-operating characteristic curve (ROC) of the nomogram and AJCC8 stage and calibration curves of the nomogram for DSS at 1, 2, and 3 years were created in the TS using the “survival” and rms packages of R v3.6.1. In addition, the net reclassification improvement (NRI) and integrated discrimination improvement (IDI) were calculated to compare the accuracy of the nomogram with that of the AJCC8 stage. After addressing the accuracy of the nomogram, the decision curve analysis (DCA) was used to compare the reliability of the nomogram and AJCC8 stage.

## Results

### Patient Demographics at Baseline and Disease Features of Patients

In the SEER cohort, 1,435 patients were selected according to the inclusion criteria and exclusion criteria. These patients were divided randomly into a TS (*n* = 1,007) and IVS (*n* = 428). Similarly, patients from two tertiary hospitals were assigned to the EVS.

In the SEER cohort, the average age at the diagnosis was 65.7 years. In the EVS, the average age at the diagnosis was 62.6 years. The median duration of follow-up (interquartile range) was 19 (11–35), 20 (12–39), and 16.6 (7.4–28.7) months for the TS, IVS, and EVS, respectively. The Kaplan–Meier method was used to calculate the survival of patients in the three groups. DSS at 3 years in the TS, IVS, and EVS was 31.9, 36.1, and 40.9%, respectively. The patient demographics and disease features of the patients are shown in Table 1.

**TABLE 1:** Baseline patient demographics and disease features in the study.

Variables	Training set (*n* = 1007)	Internal validation set (*n* = 428)	External validation set (*n* = 151)
**Age**			
<65	438 (43.5)	162 (37.9)	91 (60.3)
≥65	569 (56.5)	266 (62.1)	60 (39.7)
**Gender**			
Female	526 (52.2)	235 (54.9)	76 (50.3)
Male	481 (47.8)	193 (45.1)	75 (49.7)
**Race**			
White	776 (77.1)	330 (77.1)	*NA*
Black	113 (11.2)	55 (12.9)	*NA*
Other	118 (11.7)	43 (10.0)	151 (100)
**Site**			
Body	440 (43.7)	178 (41.6)	67 (44.4)
Tail	567 (56.3)	250 (58.4)	84 (55.6)
**Grade**			
Well	123 (12.2)	64 (15.0)	26 (17.2)
Moderate	550 (54.6)	215 (50.2)	80 (53.0)
Poor	334 (33.2)	149 (34.8)	45 (29.8)
**AJCC 8^th^**			
IA	97 (9.6)	37 (8.6)	13 (8.6)
IB	182 (18.1)	95 (22.2)	37 (24.5)
IIA	153 (15.2)	62 (14.5)	30 (19.9)
IIB	385 (38.2)	158 (36.9)	49 (32.5)
III	190 (18.9)	76 (17.8)	22 (14.6)
**T stage**			
T1	127 (12.6)	55 (12.9)	20 (13.2)
T2	418 (41.5)	200 (46.7)	62 (41.1)
T3	417 (41.4)	157 (36.7)	53 (35.1)
T4	45 (4.5)	16 (3.7)	16 (10.6)
**Radiation**			
No	656 (65.1)	263 (61.4)	117 (77.5)
Yes	351 (34.9)	173 (38.6)	34 (22.5)
**Chemotherapy**			
No	321 (31.9)	123 (28.7)	38 (25.2)
Yes	686(68,1)	305 (71.3)	113 (74.8)
**Marital status**			
Single	331 (32.9)	147 (34.3)	26 (17.2)
Married	676 (67.1)	281 (65.7)	125 (82.8)
**LNR**			
≤0.1	617 (61.3)	274 (64.0)	109 (72.2)
>0.1	390 (38.7)	154 (36.0)	42 (27.8)

*n (%) for categorical variables; LNR, Lymph nodes ratio.*

### Independent Prognostic Factors Analyzed in the TS

Based on the univariate and multivariate Cox proportional hazards regression analysis, five independent predictors were identified in the TS. Univariate Cox proportional hazards regression analysis demonstrated that sex (*p* = 0.274), tumor site (*p* = 0.452), radiotherapy (*p* = 0.705), and marital status (*p* = 0.537) had no impact upon DSS. Multivariate Cox proportional hazards regression analysis demonstrated that age ≥ 65 years at the diagnosis (HR 1.210, 95% CI 1.046–1.414, and *p* = 0.014), moderate tumor grade (2.410, 1.809–3.211, and <0.001), poor tumor grade (3.118, 2.319–4.194,and <0.001), use of chemotherapy (0.592, 0.505–0.695, and <0.001), T2 stage (1.939, 1.450–2.593, and <0.001), T3 stage (2.471, 1.845–3.307, and <0.001), T4 stage (2.766, 1.796–4.261, and <0.001), and the LNR > 0.1 (1.718, 1.476–1.999, and <0.001) were associated significantly with DSS in patients after resection of a non-metastatic APBT (Table 2). Then, these predictors were incorporated to build the nomogram.

**TABLE 2:** Cox regression of prognostic variables of the DSS in the training set.

Variables	Univariate cox		Multivariable cox	
		
	HR (95%CI)	*p-*Value	HR (95%CI)	*p*-Value
**Age**				
<65	1 (reference)		1 (reference)	
≥65	1.293 (1.114–1.501)	< 0.001	1.210 (1.046–1.414)	0.014
**Gender**				
Female	1 (reference)			
Male	1.085 (0.937–1.256)	0.274		
**Race**				
White	1 (reference)		1 (reference)	
Black	1.122 (0.894–1.409)	0.319	1.147 (0.913–1.441)	0.240
Other	0.769 (0.604–0.981)	0.034	0.834 (0.653–1.065)	0.145
**Site**				
Body	1 (reference)			
Tail	1.058 (0.913–1.226) 0.452			
**Grade**				
Well	1 (reference)		1 (reference)	
Moderate	2.378 (1.792–3.155)	<0.001	2.410 (1.809–3.211)	<0.001
Poor	3.111 (2.323–4.165)	<0.001	3.118 (2.319–4.194)	<0.001
**Radiotherapy**				
No	1 (reference)			
Yes	0.971 (0.835–1.129)	0.705		
**Chemotherapy**				
No	1 (reference)		1 (reference)	
Yes	0.722 (0.619–0.844)	<0.001	0.592 (0.505–0.695)	<0.001
**Tstage**				
T1	1 (reference)		1 (reference)	
T2	2.300 (1.725–3.067)	<0.001	1.939 (1.450–2.593)	<0.001
T3	3.027 (2.272–4.032)	<0.001	2.471 (1.845–3.307)	<0.001
T4	3.238 (2.116–4.956)	<0.001	2.766 (1.796–4.261)	<0.001
**Marital status**				
Single	1 (reference)			
Married	0.952 (0.816–1.112)	0.537		
**LNR**				
≤0.1	1 (reference)		1 (reference)	
>0.1	1.870 (1.613–2.169)	<0.001	1.718 (1.476–1.999)	<0.001

*DSS, disease-specific survival; HR, hazard ratio; CI, confidence interval; LNR, Lymph nodes ratio.*

### Nomogram Construction and Performance of the Nomogram in Stratifying the Risk of Patients

**FIGURE 2:**
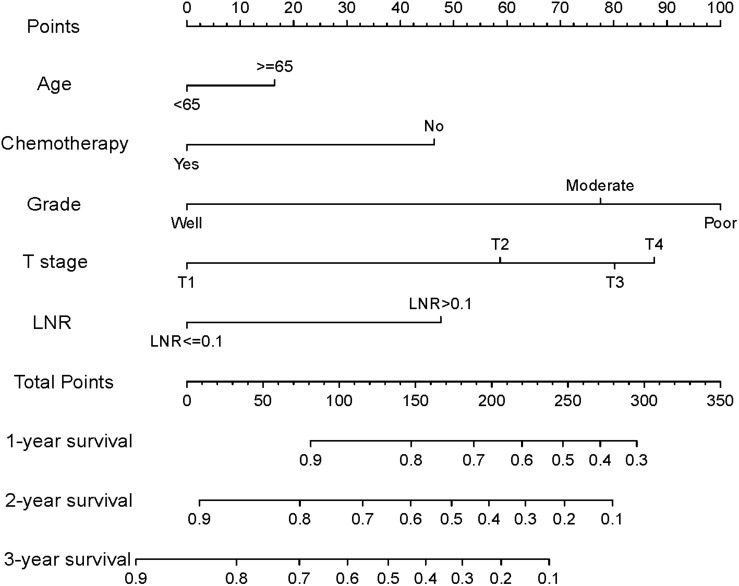
Nomogram for predicting disease-specific survival (DSS) for patients after resection of a non-metastatic adenocarcinoma of the pancreatic body and tail (age ≤ 65 years = 0 points; age > 65 years = 16; good tumor grade = 0; moderate tumor grade = 78; poor tumor grade = 100; non-use of chemotherapy = 46; use of chemotherapy = 0; T1 stage = 0; T2 stage = 59; T3 stage = 80; T4 stage = 88; the LNR ≤ 0.1 = 0; and the LNR > 0.1 = 48).

Based on the reduced multivariate models of the TS, a nomogram that combined all the independent predictors was constructed to predict DSS at 1, 2, and 3 years (Figure 2). In this model, each predictor was given a score on a point scale (age ≤ 65 years = 0 points; age > 65 years = 16; good tumor grade = 0; moderate tumor grade = 78; poor tumor grade = 100; non-use of chemotherapy = 46; use of chemotherapy = 0; T1 stage = 0; T2 stage = 59; T3 stage = 80; T4 stage = 88; the LNR ≤ 0.1 = 0; and the LNR > 0.1 = 48).

**FIGURE 3:**
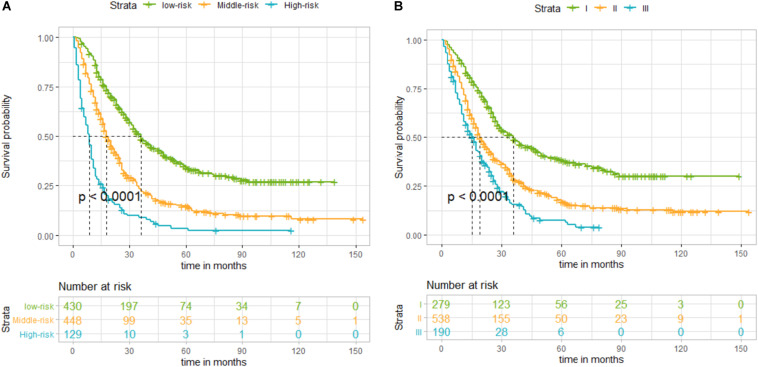
**(A)** Kaplan–Meier curves of disease-specific survival (DSS) for patients according to the risk groups of the nomogram in the training set: blue line denotes high risk, yellow line represents medium risk, and green line denotes low risk. **(B)** Kaplan–Meier curves of DSS for patients according to stages I, II, and III of the 8th edition of American Joint Committee on Cancer (AJCC8) in the training set. The blue line denotes stage III, yellow line represents stage II, and green line denotes stage I. The nomogram showed a superior ability to classify the risk stratification of patients.

By summing up the total score of each patient, we could predict the possibility of DSS at 1, 2, and 3 years. A higher total score indicated a worse prognosis. After applying cutoff values to all patients using X-tail software, the TS was grouped into three risk groups of Nomo-score: 0 ≤ low risk < 175; 175 ≤ medium risk < 244; and high-risk ≥ 244. Kaplan–Meier survival curves of DSS for the three risk groups of Nomo-score were plotted (Figure 3A). An obvious grading ability was observed in the new risk model (*p* < 0.001). To further compare the ability of the risk grading of patients, Kaplan–Meier survival curves of DSS for the different groups of the AJCC8 stage were plotted (Figure 3B). Compared with the AJCC8 stage, the nomogram showed a superior function to classify the risk stratification of patients after resection of a non-metastatic APBT.

### Calibration and Validation of the Nomogram

**FIGURE 4:**
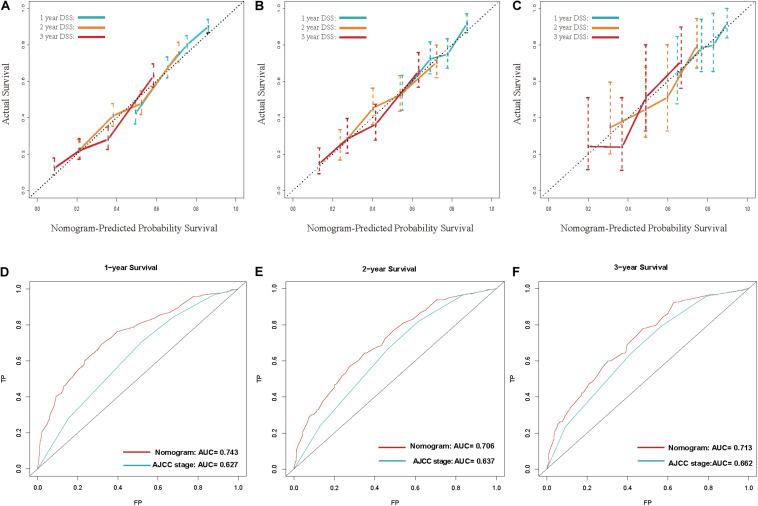
**(A–C)** Calibration plot for disease-specific survival (DSS) prediction at 1, 2, and 3 years according to the nomogram for the training set, internal-validation set, and external-validation set: the yellow line denotes 1 year DSS, green line represents 2 years, and the blue line denotes 3 years. **(D–F)** Receiver operating characteristic (ROC) curves for DSS at 1, 2, and 3 years according to the nomogram and the 8th edition of American Joint Committee on Cancer (AJCC8) staging system for the training set. For the area under the curve (AUC), the blue line denotes the AJCC8 stage, and the red line represents the nomogram. The nomogram had better accuracy for DSS prediction at 1, 2, and 3 years than that of the AJCC8 stage.

Calibration curves showed acceptable agreement between the predicted and observed values of the probability of DSS in the TS, IVS, and EVS (Figures 4A–C).

The C-index of the nomogram was 0.681 (95%CI 0.680–0.682), 0.662 (0.659–0.665), and 0.675 (0.664–0.686) in the TS, IVS, and EVS, respectively. To evaluate the discrimination ability of the nomogram, we used the AJCC8 stage to predict DSS in these patients. The C-index of the AJCC8 stage was 0.606 (95%CI 0.605–0.607), 0.590 (0.587–0.593), and 0.608 (0.596–0.620) in the TS, IVS, and EVS, respectively. The C-index of the nomogram was significantly higher than that of the AJCC8 stage in the TS, IVS, and EVS (*p* < 0.001), thereby reflecting the better overall discrimination ability of the model compared with that of the AJCC8 stage.

Next, we examined the discrimination ability of the nomogram in different years. The AUC at 1, 2, and 3 years for the nomogram was 0.743, 0.706, and 0.713, respectively, in the TS. The AUC at 1, 2, and 3 years for the AJCC8 stage was 0.627, 0.637, and 0.662, respectively, in the TS (Figures 4E–G). These data showed that the AUC values at 1, 2, and 3 years for the nomogram were all larger than that of the AJCC8 stage in the TS (*p* < 0.001). This result suggested that the nomogram had better discrimination ability than that of the AJCC8 stage for predicting DSS at 1, 2, and 3 years, respectively.

To demonstrate the accuracy of our newly built nomogram, we took advantage of the NRI and IDI. The old model was the AJCC8 stage. In the TS, the NRI for DSS at 1, 2, and 3 years was 0.535 (95% CI 0.346–0.660), 0.396 (0.249–0.529), and 0.355 (0.169–0.520), respectively. The IDI for DSS at 1, 2, and 3 years was 0.053 (*p* < 0.001), 0.070 (*p* < 0.001), and 0.066 (*p* < 0.001) in the TS, respectively. Thus, we had sufficient evidence that the nomogram had better accuracy for DSS prediction at 1, 2, and 3 years than that of the AJCC8 stage.

**FIGURE 5:**
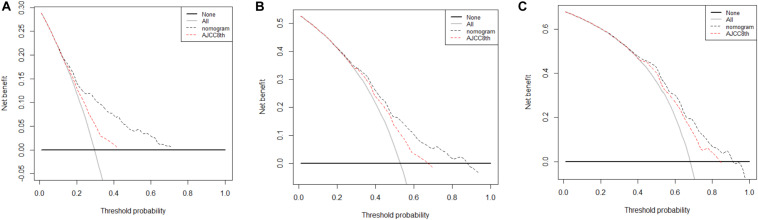
**(A–C)** Decision curve analyses of the nomogram and 8th edition of American Joint Committee on Cancer (AJCC8) stage for disease-specific survival (DSS) prediction at 1, 2, and 3 years in the training set: the red line denotes the AJCC8 stage and the black line represents the nomogram. Greater net benefits for DSS prediction at 1, 2, and 3 years were observed in the nomogram.

Finally, DCA was done to compare the clinical utility and benefits of the nomogram with that of the AJCC8 stage in the TS. DCAs at 1, 2, and 3 years of the nomogram showed larger net benefits across a range of death risks compared with that of the AJCC8 stage (Figure 5). These results confirmed that the nomogram was more practical than the AJCC8 stage.

## Discussion

Even for the small subset of patients with a pancreatic adenocarcinoma that can be resected, 5-year survival following surgery is 20% (9). An APBT has a lower possibility of resection and is associated with worse survival than that of an adenocarcinoma of the pancreatic tail (4–6). Thus, a reliable model that can be used to predict the prognosis of patients with a non-metastatic APBT is needed.

Although the AJCC8 stage is a good prognostic tool, it lacks important predictors (e.g., demographics, treatment information). To construct a more accurate prognostic model, we incorporated these factors.

We assessed a large population in the SEER database and a cohort of two tertiary hospitals in China. The age at the diagnosis, pathologic grade, use/non-use of chemotherapy, *T* stage, and the LNR were independent risk factors for patients after resection of a non-metastatic APBT. We constructed and validated a novel nomogram for predicting the DSS at 1, 2, and 3 years of these patients. We are the first research team to develop a nomogram to predict the prognosis of patients with a non-metastatic APBT.

By combining five factors of these patients, we could predict their DSS readily. Our nomogram could divide patients into three risk groups based on the total points of the five risk factors. The discrimination, calibration, and clinical meaning in this model were good. High-risk patients may have an increased risk of tumor-related death, which can guide clinicians to increase the number of reexaminations and early treatment of recurrent tumors.

The EVS from China was suitable for building a nomogram with a similar C-index. This finding was consistent with the previous result of the multivariate Cox proportional hazards regression analysis that demonstrated ethnicity was not an independent risk factor. Hence, our nomogram could be applicable across ethnicities. Interestingly, with the passage of time, the increase in AUC values, NRI, and net benefit of the nomogram were smaller relative to that in the AJCC8 stage. This result may have been due to the poor prognosis of patients with an APBT. Both models were pessimistic in terms of the long- term prognosis, so there was no obvious advantage in predicting the long-term prognosis for the nomogram compared with that using the AJCC8 stage. This result also revealed that our model may be more valuable for predicting the short-term prognosis than that using the AJCC8 stage.

Similar to previous studies on pancreatic cancer, we demonstrated that older age, higher histology grade, higher T stage, and greater LNR were poor survival factors for patients with a non-metastatic APBT after surgery (16–18). The histology grade was the strongest predictor in our study. This finding is consistent with observations by Barugola and colleagues. It confirmed that the pathologic grading was a strong predictor of early death for patients diagnosed with a pancreatic ductal adenocarcinoma who underwent pancreatectomy (19). Currently, standard treatment for patients with a resectable pancreatic tumor is resection and adjuvant multi-agent chemotherapy (gemcitabine plus capecitabine or modified FOLFIRINOX) (20). We also demonstrated that chemotherapy was a protective factor in our cohort. It is widely accepted that the LNR is an independent risk factor for the prognosis of pancreatic cancer, but the best cutoff point to predict survival is controversial (21–23). We discovered the cutoff point to be 0.1 and confirmed the LNR to be an independent risk factor. Moreover, our study confirmed that radiotherapy in these patients was not a prognostic factor for DSS, which has been a controversial issue in previous studies. Some studies have reported that adjuvant chemoradiotherapy does not benefit the survival of patients with pancreatic cancer after surgery (24–26). A phase-II clinical trial revealed that neoadjuvant FOLFIRINOX followed by individualized chemoradiotherapy enabled a high prevalence of R0 resection and prolonged survival (27). Radiotherapy delivered with protons and stereotactic body radiation may be beneficial to treatment of pancreatic cancer (28, 29). It is necessary to conduct further research on the role of radiotherapy in pancreatic cancer.

Our study had four main limitations. First, although we divided our data randomly into a TS and IVS and used a multicenter cohort of 151 patients as an EVS, a larger multicenter, prospective study is required. Second, the C-index of the nomogram was good but not perfect. Other factors, such as presence of cancer antigen 19–9, the resection margin, and physical indices (e.g., body mass index) were not available in the SEER dataset. Addition of these factors might have improved the quality of our nomogram. Third, the specific drugs used and treatment details (e.g., neoadjuvant or adjuvant chemotherapy) in the SEER database were not provided. The provision of this information could increase the predictive power of our nomogram and provide clearer clinical guidance for these patients. Finally, we did not distinguish between preoperative, intraoperative, and postoperative radiotherapy because of the insufficient sample sizes of several radiotherapy regimens. Also, the types (X-ray, protons, stereotactic body) of radiation were not stated in the SEER database. Additional, large-sample prospective research or meta-analyses should be done to assess the role of radiotherapy in this patient population.

## Conclusion

We established and validated a nomogram that provided individual predictions of DSS in patients with a non-metastatic APBT after surgery. This nomogram could be an effective tool for prognostic evaluation of these patients. However, additional external validation and a more accurate model are needed.

## Data Availability Statement

The datasets generated for this study are available on request to the corresponding author.

## Ethics Statement

The studies involving human participants were reviewed and approved by Guangdong Provincial People’s Hospital, Guangdong Academy of Medical Sciences. The patients/participants provided their written informed consent to participate in this study.

## Author Contributions

YZo, HH, and NS were responsible for the collection and analysis of data and manuscript writing. SR, LJ, and ZY analyzed and interpreted the data. HH, YZh, ZM, and ZC were in charge of data acquisition. NS, ZJ, MD, and HJ were responsible for the project development and critical revision. All authors participated in the discussion and editing of the manuscript.

## Conflict of Interest

The authors declare that the research was conducted in the absence of any commercial or financial relationships that could be construed as a potential conflict of interest.

## Footnotes

1 www.seer.cancer.gov/seerstat

2 www.r-project.org/

## References

[1] Bray F Ferlay J Soerjomtaram I Siegel R Torre L Jemal A. Global cancer statistics 2018:GLOBOCAN estimates of incidence and mortality worldwide for 36 cancers in 185 countries. *CA Cancer J Clin.* (2018) 68:394–424. 10.3322/caac.21492 30207593

[2] Siegel RL Miller KD Jemal A. Cancer statistics, 2019. *CA Cancer J Clin.* (2019) 69:7–34. 10.3322/caac.21551 30620402

[3] Stathis A Moore MJ. Advanced pancreatic carcinoma: current treatment and future challenges. *Nat Rev Clin Oncol.* (2010) 7:163–72. 10.1038/nrclinonc.2009.236 20101258

[4] Artinyan A Soriano PA Prendergast C Low T Ellenhorn JD Kim J. The anatomic location of pancreatic cancer is a prognostic factor for survival. *HPB (Oxford).* (2008) 10:371–6. 10.1080/13651820802291233 18982154 PMC2575681

[5] Ruess DA Makowiec F Chikhladze S Sick O Riediger H Hopt UT The prognostic influence of intrapancreatic tumor location on survival after resection of pancreatic ductal adenocarcinoma. *BMC Surg.* (2015) 15:123. 10.1186/s12893-015-0110-5 26615588 PMC4663036

[6] Mackay TM van Erning FN van der Geest LGM de Groot JWB Haj Mohammad N Lemmens VE Association between primary origin (head, body and tail) of metastasised pancreatic ductal adenocarcinoma and oncologic outcome: a population-based analysis. *Eur J Cancer.* (2019) 106:99–105. 10.1016/j.ejca.2018.10.008 30476732

[7] Watanabe I Sasaki S Konishi M Nakagohri T Inoue K Oda T Onset symptoms and tumor locations as prognostic factors of pancreatic cancer. *Pancreas.* (2004) 28:160–5. 10.1097/00006676-200403000-00007 15028948

[8] Dreyer SB Jamieson NB Upstill-Goddard R Bailey PJ Mckay CJ Australian Pancreatic Cancer Genome Initiative, Defining the molecular pathology of pancreatic body and tail adenocarcinoma. *Br J Surg.* (2018) 105:183–91. 10.1002/bjs.10772 29341146 PMC5817249

[9] Mizrahi JD Surana R Valle JW Shroff RT. Pancreatic cancer. *Lancet.* (2020) 395:2008–20. 10.1016/S0140-6736(20)30974-0 32593337

[10] Shi N Liu SL Li YT You L Dai MH Zhao YP. Splenic preservation versus splenectomy during distal pancreatectomy: a systematic review and meta-analysis. *Ann Surg Oncol.* (2016) 23:365–74. 10.1245/s10434-015-4870-z 26493758

[11] Kamarajah SK Burns WR Frankel TL Cho CS Nathan H. Validation of the American Joint adenocarcinoma: a surveillance, epidemiology and end results (SEER) analysis. *Ann Surg Oncol.* (2017) 24:2023–30. 10.1245/s10434-017-5810-x 28213792

[12] Allen PJ Kuk D Castillo CF Basturk O Wolfgang CL Cameron JL Multi-institutional validation study of the American Joint Commission on Cancer (8th Edition) changes for T and N staging in patients with pancreatic adenocarcinoma. *Ann Surg.* (2017) 261:185–91. 10.1097/SLA.0000000000001763 27163957 PMC5611666

[13] Balachandran VP Gonen M Smith JJ DeMatteo RP. Nomograms in oncology: more than meets the eye. *Lancet Oncol.* (2015) 16:173–80. 10.1016/S1470-2045(14)71116-7 PMC4465353 25846097

[14] Pu N Li J Xu Y Lee W Fang Y Han X Comparison of prognostic prediction between nomogram based on lymph node ratio and AJCC 8th staging system for patients with resected pancreatic head carcinoma: a SEER analysis. *Cancer Manag Res.* (2018) 10:229–38. 10.2147/CMAR.S157940 29440932 PMC5804271

[15] Li HB Zhou J Zhao FQA. prognostic nomogram for disease-specific survival in patients with pancreatic ductal adenocarcinoma of the head of the pancreas following pancreaticoduodenectomy. *Med Sci Monit.* (2018) 24:6313–21. 10.12659/MSM.909649 30198517 PMC6144730

[16] He C Zhang Y Cai Z Lin X Li S. Overall survival and cancer-specific survival in patients with surgically resected pancreatic head adenocarcinoma: a competing risk nomogram analysis. *J Cancer.* (2018) 9:3156–67. 10.7150/jca.25494 30210639 PMC6134825

[17] Li J Liu L. Overall survival in patients over 40 years old with surgically resected pancreatic carcinoma: a SEER-based nomogram analysis. *BMC Cancer.* (2019) 19:726. 10.1186/s12885-019-5958-9 31337369 PMC6651947

[18] Song W Miao DL Chen L. Nomogram for predicting survival in patients with pancreatic cancer. *Onco Targets Ther.* (2018) 11:539–45. 10.2147/OTT.S154599 29416354 PMC5790064

[19] Barugola G Partelli S Marcucci S Sartori N Capelli P Bassi C Resectable pancreatic cancer: who really benefits from resection? *Ann Surg Oncol.* (2009) 16:3316–22. 10.1245/s10434-009-0670-7 19707831

[20] Strobel O Neoptolemos J Jäger D Büchler MW. Optimizing the outcomes of pancreatic cancer surgery. *Nat Rev Clin Oncol.* (2019) 16:11–26. 10.1038/s41571-018-0112-1 30341417

[21] Zhan HX Xu JW Wang L Zhang GY Hu SY. Lymph node ratio is an independent prognostic factor for patients after resection of pancreatic cancer. *World J Surg Oncol.* (2015) 13:105. 10.1186/s12957-015-0510-0 25888902 PMC4380100

[22] Aoyama T Yamamoto N Kamiya M Murakawa M Tamagawa H Sawazaki S The lymph node ratio is an independent prognostic factor in pancreatic cancer patients who receive curative resection followed by adjuvant chemotherapy. *Anticancer Res.* (2018) 38:4877–82. 10.21873/anticanres.12801 30061263

[23] You MS Lee SH Choi YH Shin BS Paik WH Ryu JK Lymph node ratio as valuable predictor in pancreatic cancer treated with R0 resection and adjuvant treatment. *BMC Cancer.* (2019) 19:952. 10.1186/s12885-019-6193-0 31615457 PMC6794802

[24] Neoptolemos JP Stocken DD Friess H Bassi H Dunn JA Hickey H A randomized trial of chemoradiotherapy and chemotherapy after resection of pancreatic cancer. *N Engl J Med.* (2004) 350:1200–10. 10.1056/NEJMoa032295 15028824

[25] Ren F Xu YC Wang HX Tang L Ma Y. Adjuvant chemotherapy, with or without postoperative radiotherapy, for resectable advanced pancreatic adenocarcinoma: continue or stop. *Pancreatology.* (2012) 12:162–9. 10.1016/j.pan.2012.02.002 22487527

[26] Vela N Davis LE Cheng SY Hammad A Liu Y Kagedan DJ Economic analysis of adjuvant chemoradiotherapy compared with chemotherapy in resected pancreas cancer. *Ann Surg Oncol.* (2019) 26:4193–203. 31535303 10.1245/s10434-019-07808-8

[27] Murphy JE Wo JY Ryan DP Jiang W Yeap BY Drapek LC Total neoadjuvant therapy with FOLFIRINOX followed by individualized chemoradiotherapy for borderline resectable pancreatic adenocarcinoma: a phase 2 clinical trial. *JAMA Oncol.* (2018) 5:1020–7. 10.1001/jamaoncol.2018.0329 29800971 PMC6145728

[28] Nichols RC Rutenberg M. Optimizing neoadjuvant radiotherapy for resectable and borderline resectable pancreatic cancer using protons. *World J Gastrointest Surg.* (2019) 11:303–7. 10.4240/wjgs.v11.i7.303 31602289 PMC6783690

[29] Mills BN Connolly KA Ye J Murphy JD Uccello TP Han BJ Stereotactic body radiation and interleukin-12 combination therapy eradicates pancreatic tumors by repolarizing the immune microenvironment. *Cell Rep.* (2019) 29:406–21. 10.1016/j.celrep.2019.08.095 31597100 PMC6919969

